# Dr. John W. Southworth

**Published:** 1889-04

**Authors:** 


### Obituary.

DR. JOHN W. SOUTHWORTH, of Rochester, died March 17th, at the age of 46 years. During the war, he served as hospital steward, and after its close began the study of medicine, and was graduated from the University of Michigan in 1867. The early years of his professional life were spent in several places, but for the past twelve years he has practiced in Rochester. He was a member of the County Medical Society, at a meeting of which appropriate resolutions of respect and sympathy were passed.

